# Energy Metabolism Analysis of Three Different Mesenchymal Stem Cell Populations of Umbilical Cord Under Normal and Pathologic Conditions

**DOI:** 10.1007/s12015-020-09967-8

**Published:** 2020-03-17

**Authors:** Eleonora Russo, Jea-Young Lee, Hung Nguyen, Simona Corrao, Rita Anzalone, Giampiero La Rocca, Cesar V. Borlongan

**Affiliations:** 1 Department of Neurosurgery and Brain Repair, University of South Florida Morsani College of Medicine, Tampa, Florida USA; 2 Section of Histology and Embryology, Department of Biomedicine, Neurosciences and Advanced Diagnostics (BIND), University of Palermo, Palermo, Italy; 3 Department of Surgical, Oncological and Stomatological Sciences, University of Palermo, Palermo, Italy

**Keywords:** Umbilical cord mesenchymal stem cells, Wharton’s Jelly, Perivascular, Stroke, Ischemic diseases, Mitochondria, Bioenergetics, Stem cell therapy

## Abstract

Human umbilical cord mesenchymal stem cells (hUC-MSCs) are a pivotal source of therapeutically active cells for regenerative medicine due to their multipotent differentiation potential, immunomodulatory and anti-inflammatory proprieties, as well as logistical collection advantages without ethical concerns. However, it remains poorly understood whether MSCs from different compartments of the human umbilical cord are therapeutically superior than others. In this study, MSCs were isolated from Wharton’s jelly (WJ-MSCs), perivascular region (PV-MSCs) and cord lining (CL-MSCs) of hUC. These cells expressed the mesenchymal markers (CD90, CD73), stemness marker (OCT4), endothelial cell adhesion molecular marker (CD146), and the monocyte/macrophage marker (CD14) found within the MSC population implicated as a key regulator of inflammatory responses to hypoxia, was displayed by WJ-, PV-, and CL-MSCs respectively. A direct consequence of oxygen and glucose deprivation during stroke and reperfusion is impaired mitochondrial function that contributes to cellular death. Emerging findings of mitochondria transfer provide the basis for the replenishment of healthy mitochondria as a strategy for the treatment of stroke. Cell Energy Phenotype and Mito Stress tests were performed the energy metabolic profile of the three MSC populations and their mitochondrial function in both ambient and OGD cell culture conditions. PV-MSCs showed the highest mitochondrial activity. CL-MSCs were the least affected by OGD/R condition, suggesting their robust survival in ischemic environment. In this study, MSC populations in UC possess comparable metabolic capacities and good survival under normal and hypoxic conditions suggesting their potential as transplantable cells for mitochondrial-based stem cell therapy in stroke and other ischemic diseases.

## Introduction

Stroke is the second leading cause of death and disability worldwide behind heart diseases [1]. A direct consequence of oxygen and glucose deprivation (OGD) during stroke is the dysfunction of mitochondria that impairs oxidative metabolism and contributes to oxidative stress, neuronal death and inflammation [2]. Indeed, mitochondria are responsible for more than 90% of the total adenosine triphosphate (ATP) demand of the cell [3]. Accordingly, the decrease of ATP production following OGD leads to energy failure, excitotoxicity and calcium overload that, in turn, determine loss of mitochondrial membrane potential [2]. Damaged mitochondria are characterized by an increase of membrane permeability that allows the release of pro-apoptotic molecules in the cytoplasm triggering apoptotic cell death [2]. Thus, mitochondrial dysfunction plays a central role in stroke injury.

Cell-based therapies aim to replace dead cells and promote the survival of damaged cells, altogether directly aiding exogenous and endogenous repair mechanisms or indirectly, by providing trophic support and reducing inflammatory response [4, 5]. Recently, a novel therapeutic mechanism of stem cells has been demonstrated to involve the transfer of healthy mitochondria into damaged cells [6]. Mitochondria can be released through tunneling nanotubes (TNTs), microvesicles, gap junctions, cell fusion and direct uptake of isolated mitochondria [7]. Even though the signals that induce a cell to release its own mitochondria and transfer these organelles to another cell are not still clear, multiple converging lines of evidence suggests that this phenomenon can help damaged cells to recover their functions [8]. In the last few years, mitochondrial transfer has been shown to occur between several cell types, including mesenchymal stem cells (MSCs), astrocytes and neurons, and endothelial progenitor cells [9, 10]. Taken together, these observations suggest that the transfer of healthy mitochondria into damaged cells may be a novel therapeutic strategy for stroke.

Human umbilical cord (hUC)-derived MSCs (hUC-MSCs) are an enticing cellular source for regenerative medicine purposes due to their self-renewal and multipotent differentiation potential as well as to their immunomodulatory and anti-inflammatory abilities [5, 11, 12]. MSCs have been isolated from Wharton’s jelly (WJ), perivascular region (PV) and cord lining (CL) of hUC [13]. However, it is still unclear whether MSCs from a certain compartment of hUC are therapeutically superior to MSCs from other compartments [13]. Interestingly, because hUC is composed only of two arteries and a vein [13], the hUC-MSCs are physiologically adapted to survive in a relatively hypoxic and glucose-poor environment leading to the overarching hypothesis that these cells may have a therapeutic potential for the treatment of ischemic pathologies, such as stroke.

The aim of the current study was to analyze the live-cell metabolic profile and mitochondrial function of all the three hUC-MSC populations in both normal and pathological stroke conditions *in vitro*.

## Materials and Methods

### Isolation and Culture of MSCs from Different Regions of Umbilical Cord

Human umbilical cords (n = 03) were purchased from Zen-Bio and they were obtained after mothers’ informed consent, immediately after full-term births with normal vaginal delivery. The isolation of MSCs from perivascular, WJ and cord lining regions of the hUC was performed as previously described [14–16]. In particular, an enzymatic method was chosen for the isolation of PV-MSCs in order to increase the cellular harvested yield around the vessels, according to Sarugaser et al. [15]. On the other hand, an explant method was performed for the isolation of both WJ- and CL-MSCs, according to Kita et al. and Mennan et al. [14, 16]. Although the isolation methods differed, the chosen technique for harvesting MSCs from each region led to optimal cell survival and amplification across the three hUC-MSC sources. For all the three cellular types, the culture medium was consisted of DMEM low-glucose (Sigma), supplemented with 10% FBS (Gibco), 1x NEAA (Sigma) and 1x antibiotics–antimycotics (Gibco) and replaced every 2–3 days until the cells were ready for sub-culture (complete medium).

Briefly, the hUCs were washed in 1x phosphate buffered saline (PBS) in order to remove bloodstains and then rinsed in warm HBSS (Gibco) supplemented by 2x antibiotics/antimycotics (Gibco). Subsequently, the hUC was cut into pieces of about 5–6 cm length and then carefully sectioned longitudinally to expose the WJ and the blood vessels.

For the isolation of PV-MSCs, the three vessels were isolated using forceps and scalpel and placed in 40 ml of HBSS (Gibco) supplemented with 100 U/mL Type I Collagenase (Sigma) and 0.01 U/mL Hyaluronidase (Stemcell Technologies) in a 50 mL Falcon tube and left to digest in for 4 h at 37 °C. After the digestion was completed, all the vessels were removed from the suspension using forceps. The suspension, containing the cells, was centrifuged at 285 g for 10 min. Subsequently, the supernatant was discarded and the cellular pellet was treated with 50 ml of 0.8% ammonium chloride (Stemcell Technologies) and incubated at room temperature for 5 min to lyse the erythrocytes. Thereafter, the tube was centrifuged for 10 min at 285 g and the supernatant was discarded. The cells, obtained from the perivascular region of each hUC, were counted and plated in one non-coated T-75 tissue culture flask with complete medium and put it in 5% CO_2_ incubator at 37 °C.

For isolation of WJ-MSCs and CL-MSCs, after removing the vessels, the remaining cord was cut in smaller pieces with a length of about 1.5 cm and each piece was placed in a well of a non-coated 6-well culture plate with complete medium and incubated overnight in 5% CO_2_ at 37 °C. The following day, the WJ absorbed the complete medium containing phenol red and, thus, it can be distinguished from the CL that is about 1 mm width. The WJ was separated from the CL from each piece of hUC by using a scalpel. The collected WJ was plated in a well of a non-coated 6-well culture plate and each piece of the remaining CL was plated in a well of another non-coated 6-well plate with the sub-amnion side touching the bottom of the well to allow the sub-amnion cellular exit and attachment to the culture plate. The WJ and CL were cultured with complete medium changed every 2–3 days over for 15 days in culture, and the cellular exit from both tissues, as well as the attachment to the plastic surface of the tissue culture plate, was monitored by phase-contrast microscopy. After 15 days, the WJ and CL tissues were removed and the attached cells were cultured until reaching the confluence.

The isolated cells were subcultured until passage 4 for all the three cellular types. Morphological analysis was performed by observing the cells at the phase contrast microscope Olympus. For all subsequent experiments, a minimum of three independent replicates was done.

### Immunocytochemistry/ IF

Cells were seeded at the density of 1 × 10^4^ cells /cm^2^ in 8-well chamber slides and cultured for 2 days. Subsequently the cells were fixed with 4% paraformaldehyde in PBS for 20 min at room temperature. After a wash with PBS, 0.1% Triton X-100 (w/v) in PBS was added and kept incubated for 5 min to allow the permeabilization. After rinsing with PBS, nonspecific binding was blocked by incubating 3% normal goat serum (Invitrogen 50-062Z) in 0.1% Triton-X100 in PBS for 1 h at room temperature. Each primary antibody (Ab) was diluted in blocking buffer and incubated with samples overnight at 4 °C. Dilution of each primary Ab is specified in Table 1. After 3 washes (5 min for each wash) with PBS, secondary Abs were added for 1 h at room temperature (Table 1). Next, additional 3 washes were performed, each 5 min with PBS, then the slides were mounted with Antifade Mounting Medium with DAPI (Vectashield HardSet H-1500) and fluorescent images were collected using a Zeiss microscope. Three independent observers evaluated the immunohistochemical results and quantified the percentage of positive cells for each marker.

**Table 1:** Antibodies used for the immunofluorescence staining

Antigen	Host	Clonality	Supplier	Code	Dilution
CD90	Rabbit	Monoclonal	Abcam	AB133350	1:100
CD73	Mouse	Monoclonal	Abcam	AB54217	1:500
Oct4	Rabbit	Polyclonal	Abcam	AB19857	1: 400
CD146	Rabbit	Monoclonal	Abcam	AB75769	1: 100
CD14	Mouse	Monoclonal	Abcam	AB181470	1: 100
Goat anti-mouse IgG H&L alexa fluor 488	Goat	Polyclonal	Abcam	AB150117	1: 500
Goat anti-rabbit IgG H&L alexa fluor 594	Goat	Polyclonal	Abcam	AB150080	1: 500

### Oxygen-Glucose Deprivation/Reperfusion

PV-, WJ- and CL-MSCs were seeded at the density of 10,000 cells/ cm^2^ and cultured until reaching confluence. The cells were exposed to OGD/R to mimic the ischemic and the reperfusion condition following stroke as previously described [17]. Briefly, after the removal of the growth medium, the cells were exposed to PBS and placed in an anaerobic chamber (Plas Labs) containing nitrogen (95% N_2_) and carbon dioxide (5% CO_2_) for 15 min at 37 °C. Finally, the chamber was sealed and incubated for 90 min at 37 °C (hypoxic–ischemic condition). OGD was terminated by removal of PBS, and addition of the growth medium, then the cell cultures were then reintroduced to the regular 95% O_2_ and 5% CO_2_ incubator (normoxic condition) at 37 °C for 24 h, which represented a model of “reperfusion.”

### Measurement of Cell Viability

Measurement of cell viability was performed using fluorescent live cell assay. Both control and OGD/R treated cells were incubated with 1 µM Calcein-AM (Trevigen) for 30 min in the regular 95% O_2_ and 5% CO_2_ incubator at 37 °C. The green fluorescence of the live cells was measured by the EnSpire Multimode Plate Reader (Ex/Em = 490/520; Perkin Elmer). In addition, a morphological analysis was performed using a phase contrast microscope (Olympus).

### Cell Energy Phenotype Test

PV-, WJ- and CL-MSCs were seeded into the Seahorse XF96 Cell Culture Microplates (Agilent) at the cell density of 10,000 cells/cm^2^ and cultured until confluence. Prior to the start of the Seahorse XF Cell Energy Phenotype test, a sensor cartridge was hydrated in Seahorse XF Calibrant (Agilent) following the manufacturer’s instruction (Agilent). On the day of the assay, the cells were washed once and incubated in XF Seahorse Base Medium DMEM (Agilent) supplemented with 10 mM glucose, 1 mM sodium pyruvate, and 2 mM L-glutamine. The XF Seahorse Base Medium DMEM was prepared following the manufacturer’s instruction (Agilent), but with slight modification. Although the Agilent protocol suggests two washes, the cells were rinsed only one time to avoid the risk of detaching the cells from the plate. After calibration of the Seahorse XF96 Analyzer (Agilent), the sensor cartridge was removed from the instrument and Seahorse XF96 Cell Culture Microplate was inserted. For all the three types of cells, the oxygen consumption rate (OCR) and the extracellular acidification rate (ECAR) readings were taken over time under basal conditions and after the addition of mitochondrial inhibitors Oligomycin (1 µM) and carbonilcyanide p-triflouromethoxyphenylhydrazone (FCCP, 1 µM). With the simultaneous injection of these stressor compounds two events occurred: Oligomycin inhibited ATP production by the mitochondria and, consequently, there was a compensatory increase in the rate of glycolysis. FCCP depolarized the mitochondrial membrane that increased the OCR because the mitochondria attempted to restore the mitochondrial membrane potential.

In addition, Hoechst 33342 (Fluka, Biochemika) at the concentration of 1:3000/well was added and the absorbance was measured with EnSpire Multimode Plate Reader (Ex/Em = 358/461 nm; Perkin Elmer) for the normalization of the Seahorse data. The total run was 1 h and 12 min and the run protocol is described in Table 2. Results were exported by using the Seahorse Wave 2.4 XF-96 software and the data obtained were normalized to the absorbance per well and expressed in pmol/min/abs.

**Table 2:** Seahorse XF cell phenotype test run protocol

Command	Time (min)	Port	Drug
Calibrate			
Equilibrate	12		
Mix (×5)*	3		
Measure (×5)*	3		
Inject		A	Oligomycin + FCCP
Mix (×5)**	3		
Measure (×5)**	3		
Inject		B	Hoechst 33342
End protocol			

*After the equilibration, the baseline step consisted of 5 “mix and measure” cycles**After the injection of Oligomycin + FCCP, 5 “mix and measure” cycles were performed

### Cell Mito Stress Test

PV-, WJ- and CL-MSCs were seeded into the Seahorse XF96 Cell Culture Microplates (Agilent) at the cell density of 10,000 cells/cm^2^ and cultured until confluence. The cells were exposed to OGD/R as described above. Prior to the start of the Seahorse XF Cell Energy Phenotype test, a sensor cartridge was hydrated in Seahorse XF Calibrant (Agilent) following the manufacturer’s instruction (Agilent). The day of the assay, the cells were washed one time and incubated in XF Seahorse Base Medium DMEM (Agilent) supplemented with 5.5 mM glucose, 1 mM sodium pyruvate, and 4 mM L-glutamine and 1x NEAA to mimic both cell growth and reperfusion conditions after OGD. The XF Seahorse Base Medium DMEM was prepared following the manufacturer’s instruction (Agilent). For both control and OGD/R conditions, OCR and ECAR readings were taken over time under basal conditions and after the addition of mitochondrial inhibitors (1 µM oligomycin, 1 µM FCCP and 0.5 µM rotenone/antimycin).

In addition, Hoechst 33342 (Fluka, Biochemika) was added at the concentration of 1:3000/well. The absorbance was measured with EnSpire Multimode Plate Reader (Ex/Em = 358/461 nm; Perkin Elmer) and the nuclei count was performed with a fluorescent phase contrast microscope (Olympus) for the normalization of the Seahorse data.

The total run was 1 h and 24 min and the run protocol is described in Table 3. Results were exported by using the Seahorse Wave 2.4 XF-96 software and the data obtained for each condition were normalized to the cell number per well and expressed in pmol/min/cells.

**Table 3:** Seahorse XF cell mito stress test run protocol

Command	Time (min)	Port	Drug
Calibrate			
Equilibrate	12		
Mix (x 3)*	3		
Measure (x3)*	3		
Inject		A	Oligomycin
Mix (x3)**	3		
Measure (x3)**	3		
Inject		B	FCCP
Mix (x3)**	3		
Measure (x3)**	3		
Inject		C	Rotenone/Antimycin
Mix (x3)**	3		
Measure (x3)**	3		
Inject		D	Hoechst 33342
End protocol			

*After the equilibration, the baseline step consisted of 3 “mix and measure” cycles

**After the injection of Oligomycin, FCCP and Rotenone/Antimycin follow 3 “mix and measure” cycles

### Statistical Analysis

All statistic analyses were performed using GraphPad Prism software (https://www.graphpad.com/scientific-software/prism/). Data are shown as mean ± SEM. Statistical analysis was performed with either Mann Whitney or Wilcoxon tests.

## Results

### Characterization of the Three MSCs Populations of UC

**Fig. 1:**
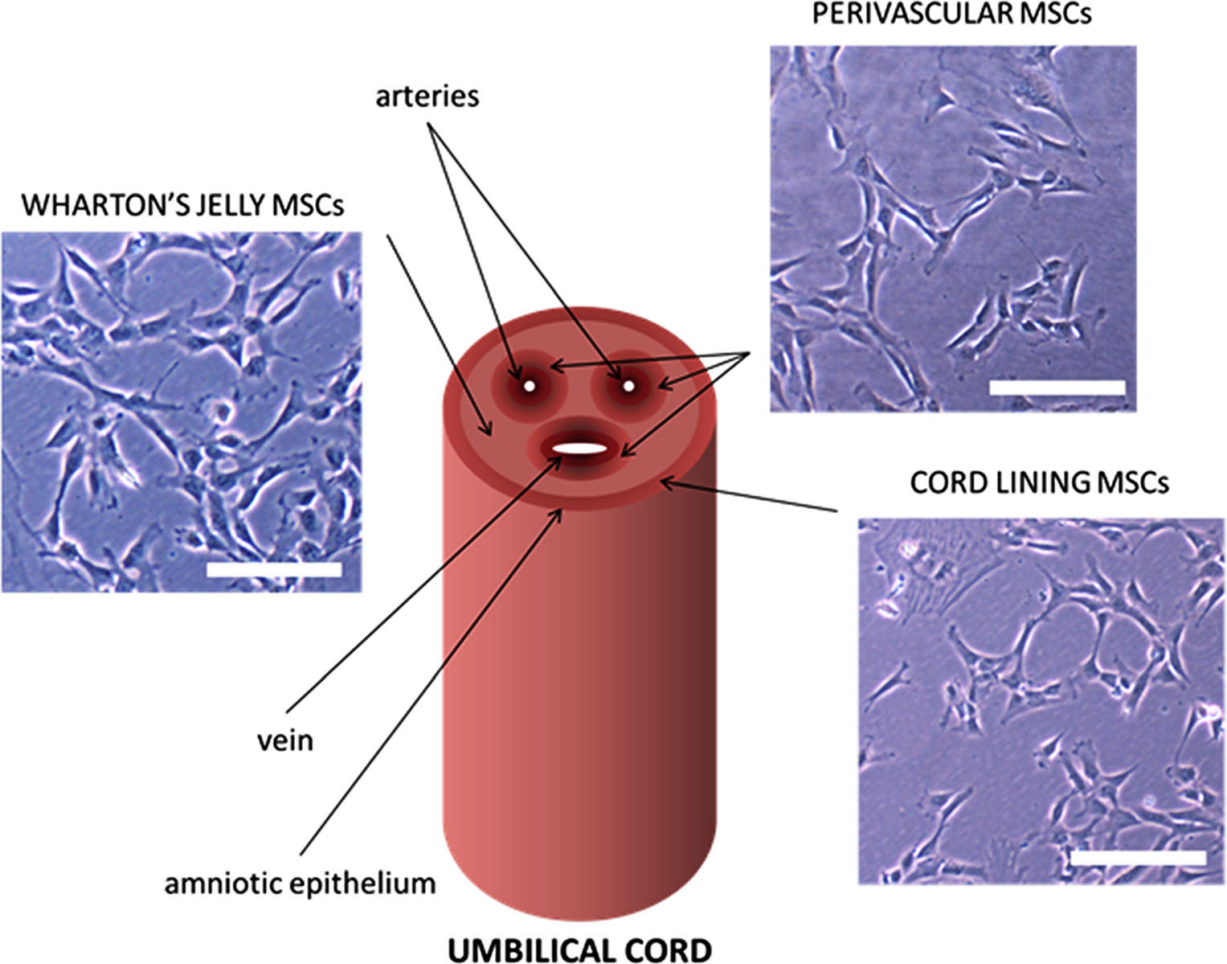
Morphological features of the three MSC populations of the UC at P0. Magnification 20x, bar 50 µm

**Fig. 2:**
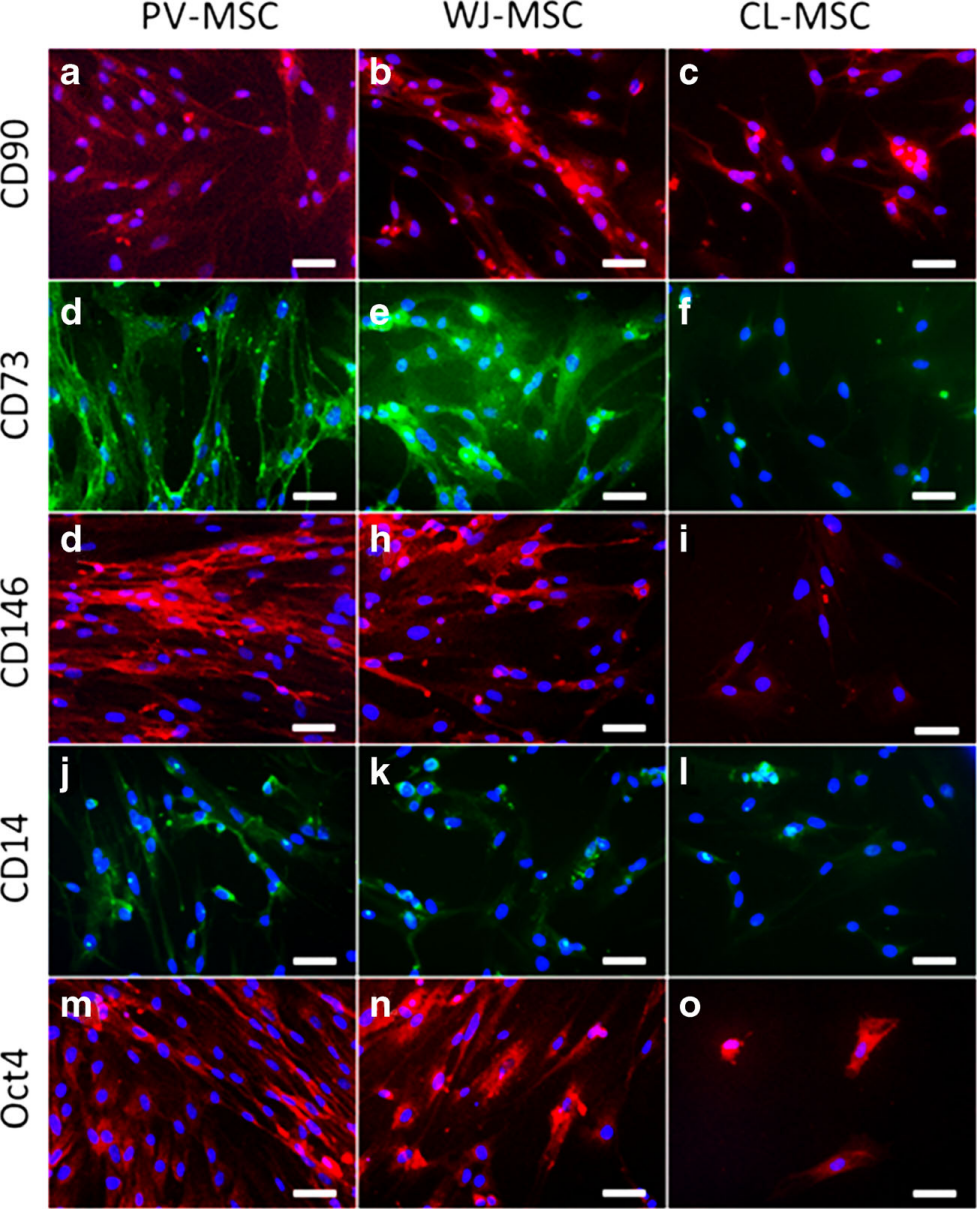
Immunolocalization of CD90, CD73, Oct4, CD146 and CD14. Magnification 20x, bar 50 µm. Immunofluorescence staining reveals that the cells all expressed MSC markers (CD90 and CD73). In addition, PV-, WJ- and CL-MSCs expressed CD146, Oct4 and CD14, respectively. The nuclei were stained with DAPI

In view of accumulating evidence of the existence of three different populations of MSCs in hUC as well as the necessity of a phenotypic characterization with a greater clarity of their properties, we isolated PV-, WJ- and CL-MSCs from UC as described in the Methods section. The number of cells obtained at P0 from the tested hUCs is shown in Table 4. The morphological analysis is shown in Fig. 1. Immunofluorescence staining showed that they all expressed mesenchymal markers CD90 and CD73 (Fig. 2). In addition, PV-MSCs expressed CD146, WJ-MSCs expressed Oct4 and CL-MSCs expressed CD14 (Fig. 2), according with previous findings by our group and others 2012 [15, 16, 18, 19]. In addition, the results of the immunocytochemical analysis are represented semiquantitatively in Table 5.

**Table 4:** Number of cells at P0 at confluence

	Cord 1	Cord 2	Cord 3
PV-MSCs	4.12 × 10^6^	2.18 × 10^6^	2.075 × 10^6^
WJ-MSCs	10.7 × 10^6^	14.55 × 10^6^	4.65 × 10^6^
CL-MSCs	9.8 × 10^6^	7.95 × 10^6^	12.5 × 10^6^

**Table 5:** Immunocytochemistry results of markers expression

	CD90	CD73	CD146	Oct4	CD14
PV-MSC	++	+++	+++	+++	+++
WJ-MSC	+++	++	+++	+++	++
CL-MSC	+++	++	+++	++	++

Results of the immunocytochemical analysis are represented semiquantitatively. Frequency of cells (#): + = # < 33%; ++ = 33 % < # < 66 %; +++ = # > 66%)

### Mitochondrial Function of PV-, WJ- and CL-MSCs

In order to understand the metabolic energy profile and mitochondrial function of PV-, WJ- and CL-MSCs, Cell Energy Phenotype and Cell Mito Stress tests were performed by using the Seahorse XF96 Analyzer. Our overall goal was to analyze the metabolic energy profile of the three hUC-MSCs populations.

**Fig. 3:**
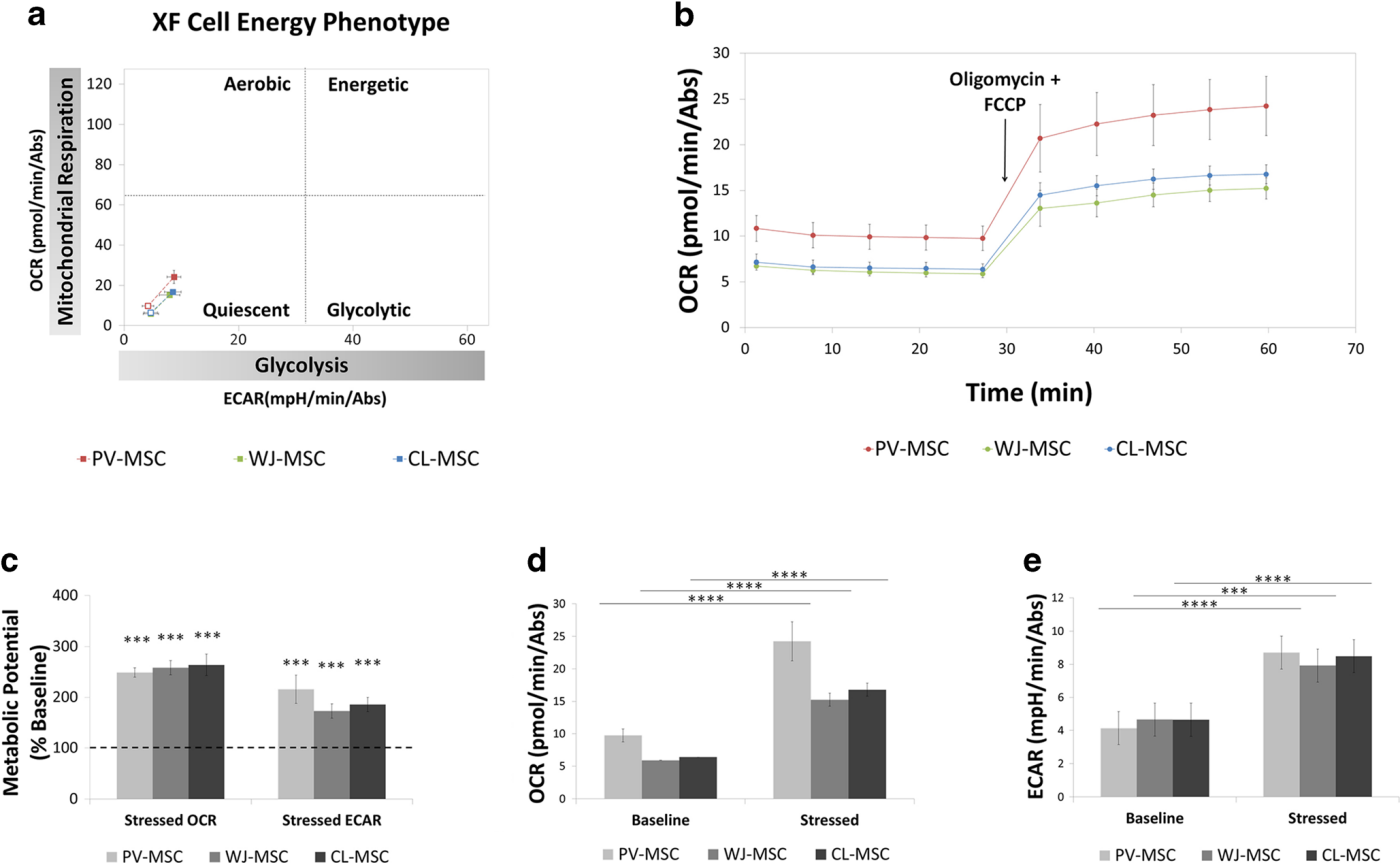
Seahorse XF Cell Energy Phenotype Test performed by using a Seahorse XF96 Analyzer (n = 12 for each cell type). Oligomycin 1 µM, FCCP 1 µM. OCR: oxygen consumption rate. ECAR: extracellular acidification rate. *** P < 0.001; **** P < 0.0001 vs. Baseline

The XF Cell Energy Phenotype Test (Fig. 3) revealed that all three hUC-MSCs populations displayed a comparable metabolic phenotype with significant increases of metabolic response with respect to baseline (Fig. 3C, p = 0.0005; Fig. 3D, PV-MSC p < 0.0001, WJ-MSC p < 0.0001, CL-MSC p < 0.0001; Fig. 3E, PV-MSC p < 0.0001, WJ-MSC p = 0.0001, CL-MSC p < 0.0001 ). In addition, our data suggest that the metabolic profile of PV-, WJ- and CL-MSCs can be classified as “quiescent”. This is evidenced by undifferentiated MSCs exhibiting low levels of mitochondrial activities and low levels of glycolytic activities [20].

**Fig. 4:**
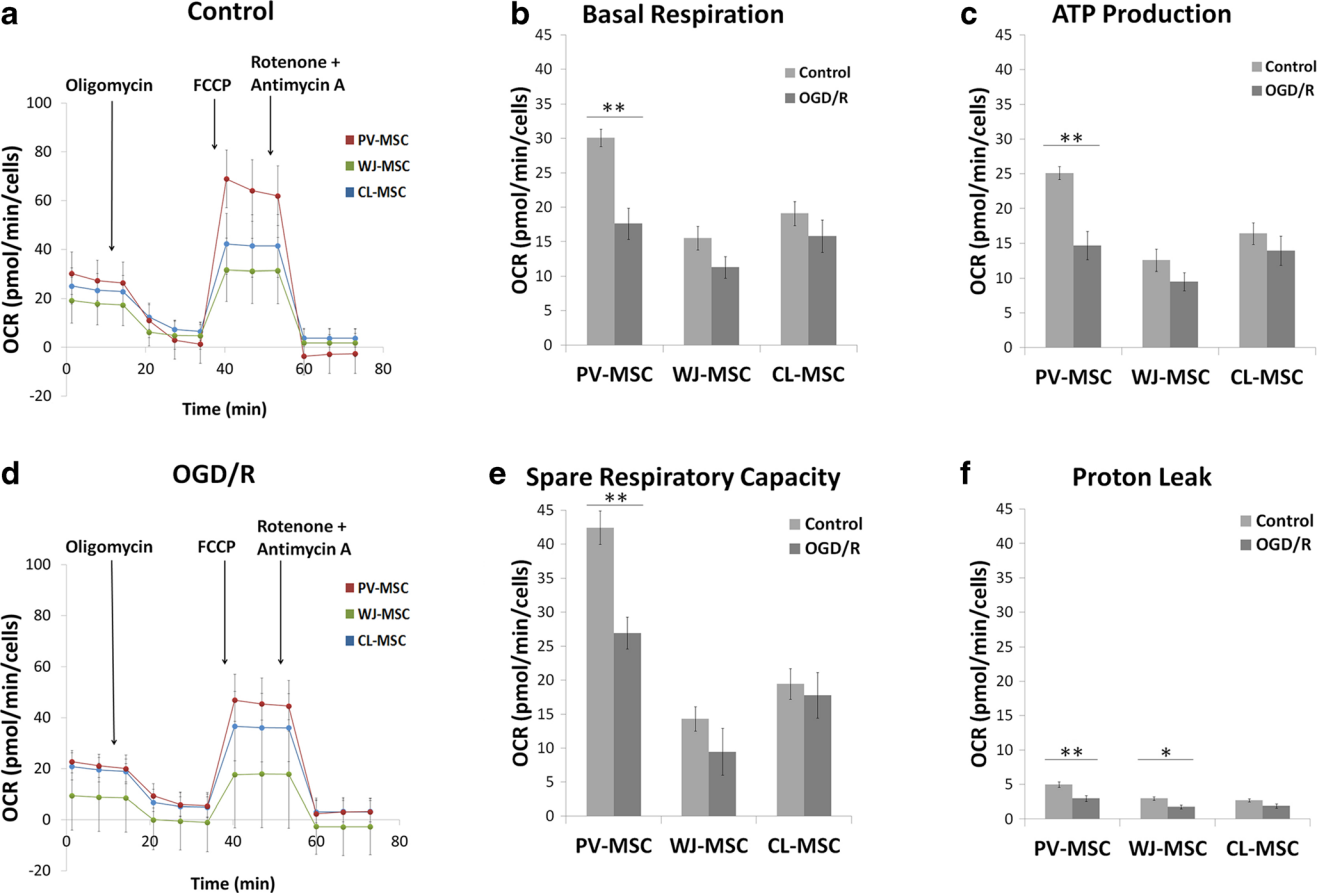
Seahorse XF Mito Stress test shows PV-, WJ- and CL-MSCs in both normal and after OGD/R conditions (n = 07 for PV-MSC and CL-MSC; n = 08 for WJ-MSC control; n = 06 for WJ-MSC OGD/R). OCR: oxygen consumption rate. 1 µM Oligomycin, 1 µM FCCP, 0.5 µM Rotenone + Antimycin A. **P < 0.01; *P < 0.05

In order to assay the mitochondrial function of PV-, WJ- and CL-MSCs in ischemic/reperfused conditions, a Cell Mito Stress test was performed on both controls and cells subjected to OGD/R (Fig. 4). Seahorse XF Cell Mito Stress test showed that all three hUC-MSCs populations exhibited comparable mitochondrial respiration parameters in both control and OGD/R conditions, demonstrating their ability to survive in ischemic/reperfused conditions (Fig. 4B, PV-MSC p = 0.0041; Fig. 4C, PV-MSC p = 0.0023; Fig. 4E PV-MSC p = 0.0012; Fig. 4F, PV-MSC p = 0.0070, WJ-MSC p = 0.0293).

**Fig. 5:**
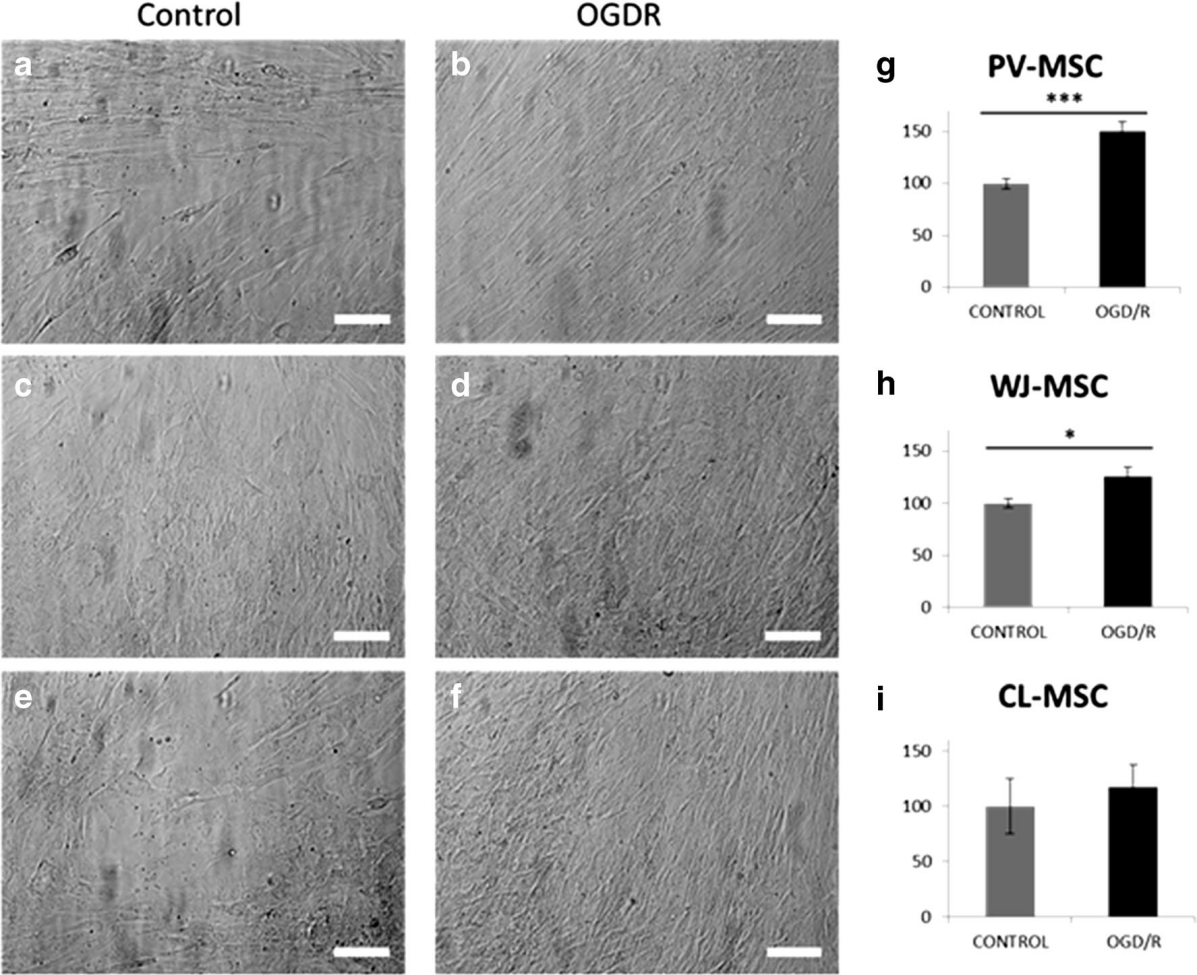
Cell viability tested by using Calcein AM stain in both control and after OGD/R conditions (n = 21 for each cell type). Magnification 10x, bar 100 µM. ***P < 0.001; *P < 0.05

In addition, a cell viability test showed that all three hUC-MSCs cellular populations showed good survival and maintained their proliferation following OGD/R, further suggesting their ability to survive in ischemic/reperfused conditions (Fig. 5, PV-MSC p = 0.0005; WJ-MSC p = 0.0198). These results demonstrate the adaptive capacity of PV-, WJ- and CL-MSCs under OGD/R conditions. This capacity to survive is probably due to their robust mitochondrial function. In particular, the OGD/R condition did not affect WJ-MSC and CL-MSC metabolism and viability (Figs. 4 and 5). Although OGD/R condition significantly altered PV-MSC metabolism, their cell viability remained robust (Figs. 4 and 5). In summary, the three populations of hUC-MSCs could be a potential source of mitochondria-based stem cell therapy for stroke.

## Discussion

Mitochondria dysfunction is a direct consequence of oxygen and glucose deprivation during stroke that contributes to oxidative stress, neuronal death and inflammation [2]. Emerging evidence of mitochondria transfer from stem cells to ischemic cells paved the way for mitochondrial-based stem cell therapy of stroke [6, 7, 10]. hUCs received only three blood vessels suggesting that hUC-MSCs are normally adapted to survive in a relatively hypoxic and glucose-poor environment, thus these cells represent an attractive source for stem cell-based therapy in ischemic pathologies, such as stroke [13]. Although several studies have shown that the regulation of energy metabolism is critical for MSC functions and their proliferation and differentiation dynamics, few studies have been focused on the energy metabolism and mitochondrial function of hUC-MSCs [20–22]. In addition, growing evidence suggests the existence of three different populations of hUC-MSCs (PV-, WJ- and CL-MSCs) [13]. However, whether a population is superior to another is not still clear [23]. Likewise, whether the different anatomical distance from the vessels of the three MSCs population in the UC might result in a different energy metabolism and in a growing resistance and survival capacity in a poor oxygen and glucose environment has not been investigated.

With this gap in knowledge, PV-, WJ- and CL-MSCs were isolated from hUCs in order to understand (1) the energy metabolism profile, (2) the mitochondrial function and (3) their survival capacity in both normal and after ischemic/reperfused conditions. Studies in bone marrow-derived -MSCs showed that these cells display a glycolytic metabolism in undifferentiated state, while a switch to OXPHOS metabolism occurred during the differentiation [20]. These previous studies were performed using different methods including measurement of mitochondrial mass, determination of mtDNA copy number, western blot analysis of glycolytic and mitochondrial enzymes, assay of the expression of mitochondrial biogenesis-associated genes, assessment of intracellular ATP content, OCR measurement using the 782 OxygenMeter and quantization of radioactive labeled glucose [20, 24, 25]. Few studies have used the Seahorse analyzer for OCR measurement in differentiated AD-MSCs and iPSC-derived mesenchymal progenitor cells [26–28]. A study analyzed the hUC-MSCs metabolism by measuring the dissolved O_2_ and pH values in the culture medium using the SFR-Shake Flask Reader [21].

Here, for the first time, the energy metabolism of the three populations of hUC-MSCs was analyzed by using the Seahorse Analyzer, which allows a sensitive kinetic measure of OCR, and ECAR in live cells and in real time. OCR is the rate of the decrease of oxygen concentration in the assay medium and, therefore, it is an indicator of mitochondrial respiration. ECAR corresponds to the rate of increase in proton concentration (or decrease in pH) in the assay medium and, therefore, it is a measure of the rate of glycolysis. In this study, the cell energy phenotype test and cell mito stress test assays were performed by using the Seahorse Analyzer (Figs. 3 and 4). The results of the cell energy phenotype test showed that PV-, WJ- and CL-MSCs are characterized by a similar energy metabolism (Fig. 3). In particular, the three MSC types exhibited a quiescent phenotype. Thus, PV-, WJ- and CL-MSCs maintained both mitochondrial and glycolytic activities at low levels.

Under stress conditions (Oligomycin + FCCP injection) the three types of MSCs displayed an increase in the glycolytic pathway likely to balance the reduction of mitochondrial respiration. Moreover, the Seahorse cell mito stress test was performed in both normal and after OGD/R to analyze the mitochondrial activity of PV-, WJ- and CL-MSCs in ischemic/reperfused conditions (Fig. 4). Although the OCR exhibited a slight decrease in OGD/R groups, all the three hUC-MSC populations showed a comparable robust resistance and adaption to ischemic/reperfused conditions as also demonstrated by the cell viability test. Indeed, the survival of cells was not affected by OGD/R treatment but surprisingly the number of the cells increased suggesting a powerful ability of all three types of hUC-MSCs to survive in stroke conditions (Fig. 5).

That MSCs augment mitochondria function may play a key role in sequestering the mitochondrial dysfunction implicated in stroke-induced secondary cell death [29–31]. The isolation of potent hUC-MSCs [32–39] towards repairing the mitochondria stands as novel stroke therapy. Cell-based regenerative medicine, which has been demonstrated to be a potent treatment for a number of neurological disorders [39–46], may benefit from transplantation of stem cell-derived mitochondria.

Taken together, these results demonstrate the adaptive capacity of PV-, WJ- and CL-MSCs to ischemic environments due to their maintained mitochondrial function. All three types of hUC-MSCs displayed a similar energy metabolism and mitochondrial function. PV-MSCs showed the highest OCR values in both Seahorse tests suggesting a superior mitochondrial activity in these cells compared to the other hUC-MSC populations. A further support of this result is the more consistent reduction of OCR of PV-MSCs after OGD/R compared to WJ- and CL-MSCs (Fig. 4). CL-MSCs were the cells least affected by OGD/R condition (Fig. 4), suggesting their robust survival in ischemic environment. Further investigations are needed to better understand whether these slight but significant differences among the three hUC-MSCs are due to the specific region’s composition of different number of healthy mitochondria or improved adaptation of mitochondria to ischemic conditions. Despite these limitations, hUC-MSCs appear to tolerate the non-conducive cellular environment and continue to display viable and functional mitochondriaafter ischemic/reperfusion injury. Thus, the three populations of hUC-MSCs stand as promising cell source for mitochondria-based stem cell therapy of stroke.

## Conclusions

Energy metabolism assay via the Seahorse technology revealed for the first time that PV- MSCs, WJ- MSCs, and CL-MSCs of the hUC displayed a robust mitochondrial profile with great capacity of survival under ischemic conditions suggesting that these hUC-MSCs represent an effective source of donor cells for mitochondria-based stem cell therapy in stroke and likely other ischemic disorders.
